# Supplementary material for: Morphological Characteristics and Expression Patterns of CmCYC2c of Different Flower Shapes in Chrysanthemum morifolium
Source: Plants (Basel). 2023 Oct 30;12(21):3728. doi: 10.3390/plants12213728 (PMC10647454; doi:10.3390/plants12213728)
Supplement: Supplementary file 1 [file plants-12-03728-s001.zip › supplementary Figures-10.6.pptx]

## Slide 1
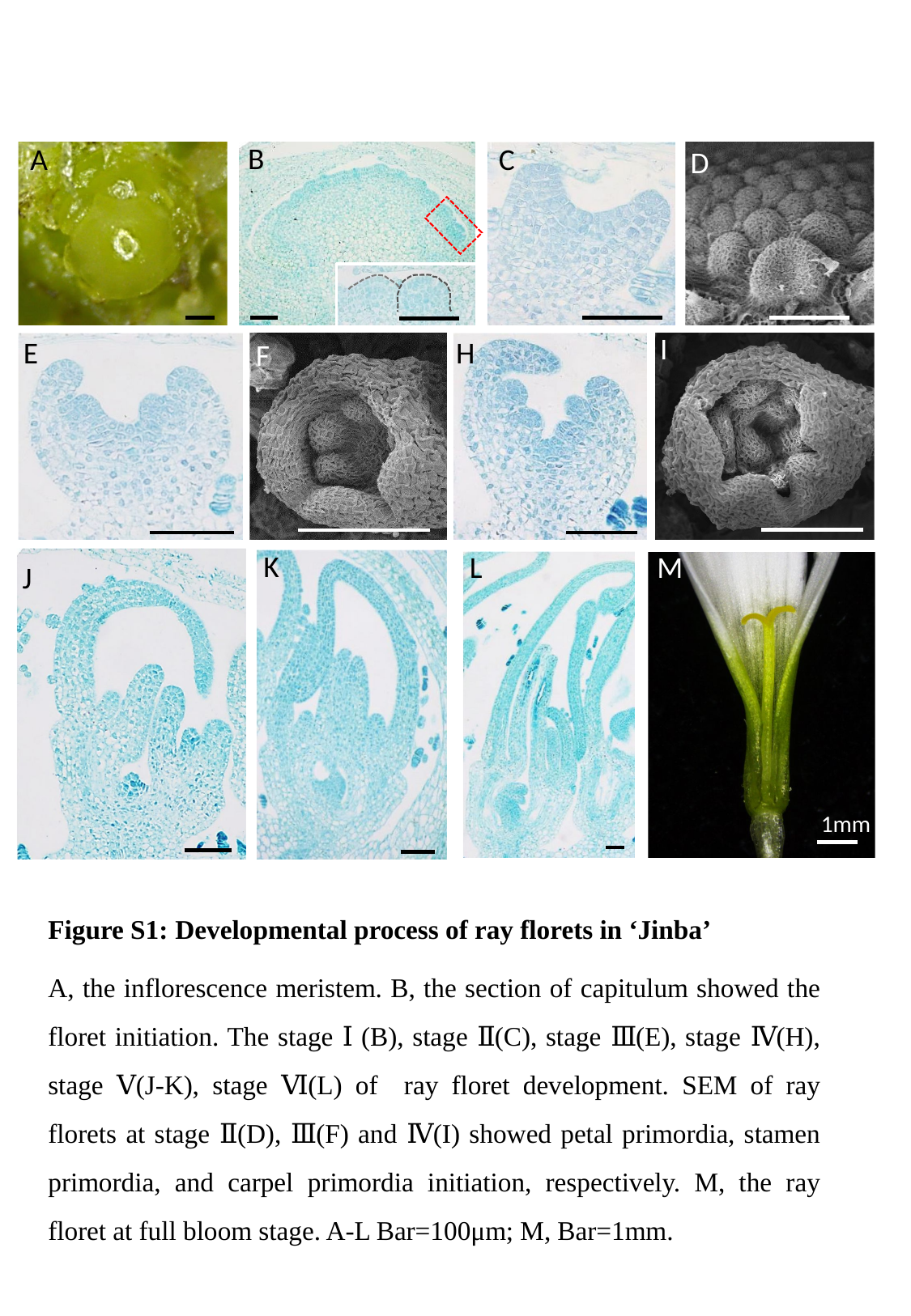

B
A
C
D
1mm
I
E
H
F
K
L
M
J
Figure S1: Developmental process of ray florets in ‘Jinba’
A, the inflorescence meristem. B, the section of capitulum showed the floret initiation. The stage Ⅰ (B), stage Ⅱ(C), stage Ⅲ(E), stage Ⅳ(H), stage Ⅴ(J-K), stage Ⅵ(L) of ray floret development. SEM of ray florets at stage Ⅱ(D), Ⅲ(F) and Ⅳ(I) showed petal primordia, stamen primordia, and carpel primordia initiation, respectively. M, the ray floret at full bloom stage. A-L Bar=100μm; M, Bar=1mm.

## Slide 2
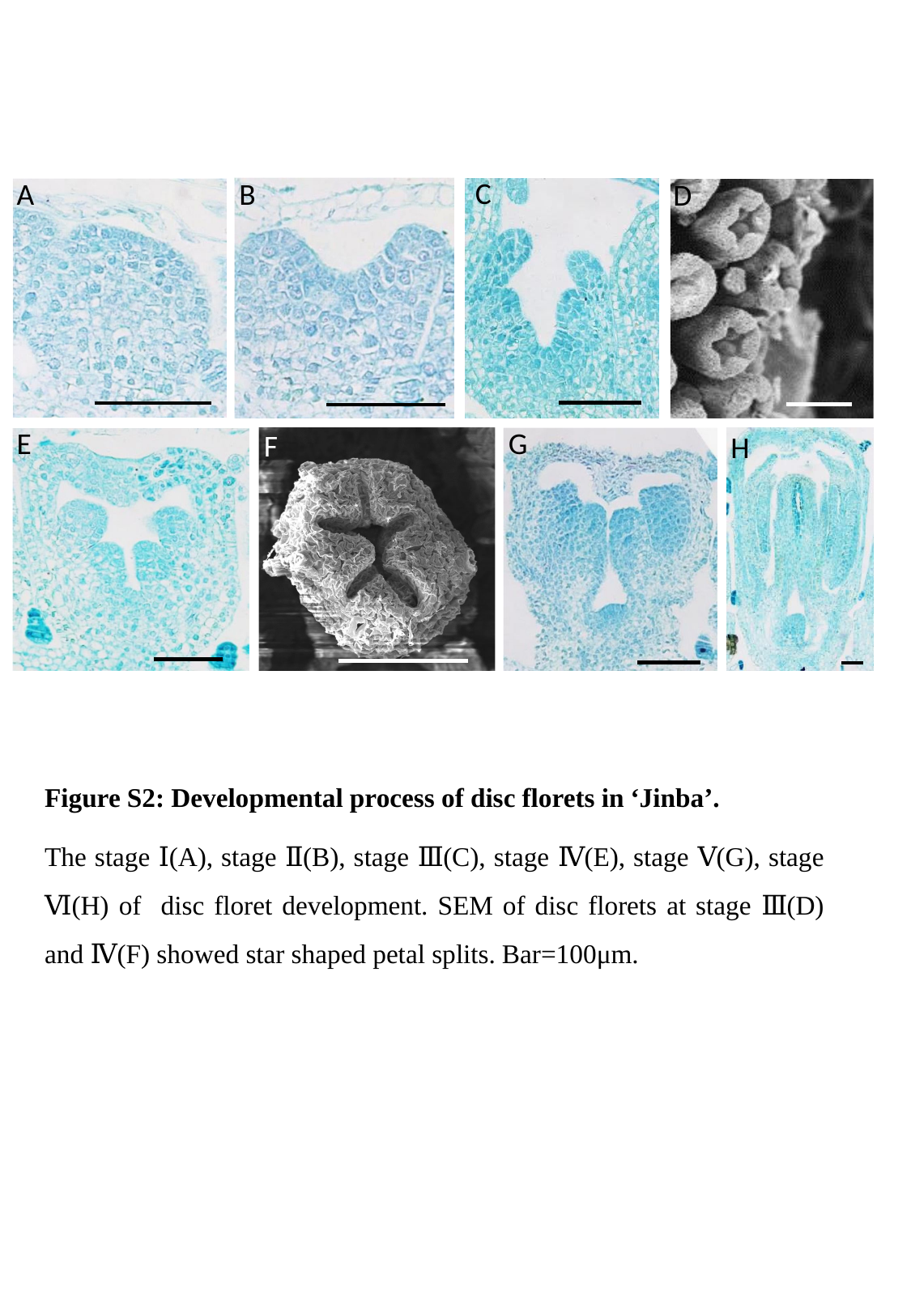

C
A
B
D
E
G
F
H
Figure S2: Developmental process of disc florets in ‘Jinba’.
The stage Ⅰ(A), stage Ⅱ(B), stage Ⅲ(C), stage Ⅳ(E), stage Ⅴ(G), stage Ⅵ(H) of disc floret development. SEM of disc florets at stage Ⅲ(D) and Ⅳ(F) showed star shaped petal splits. Bar=100μm.
