# Supplementary material for: Morphological Characteristics and Expression Patterns of CmCYC2c of Different Flower Shapes in Chrysanthemum morifolium
Source: Plants (Basel). 2023 Oct 30;12(21):3728. doi: 10.3390/plants12213728 (PMC10647454; doi:10.3390/plants12213728)
Supplement: Supplementary file 1 [file plants-12-03728-s001.zip › table S1.docx]

**Table S1. Primer sequences used in this study**

| **Primers for in situ hybridization** | |
| --- | --- |
| CmCYC2c-SP6 | GATTTAGGTGACACTATAGaatGCTACAAAGCAAGCAAAACCCTTG |
| CmCYC2c-T7 | tgTAATACGACTCACTATAGGGCACCATTAACTCCTGCCCAGA |
